# Association between congenital Zika syndrome and hospitalizations during early childhood: a nationwide cohort study

**DOI:** 10.1016/j.ijid.2025.107780

**Published:** 2025-04

**Authors:** João Guilherme G. Tedde, Thiago Cerqueira Silva, Laura Rodrigues, Maria da Conceição Costa, Luciana Cardim, Elizabeth B. Brickley, Maria Gloria Teixeira, Mauricio L. Barreto, Enny S. Paixão

**Affiliations:** 1 Center for Data and Knowledge Integration for Health (CIDACS), Oswaldo Cruz Foundation, Salvador, Brazil; 2 Faculty of Epidemiology and Population Health, London School of Hygiene & Tropical Medicine, London, UK; 3 Institute of Collective Health (ISC), Federal University of Bahia, Brazil

**Keywords:** Zika virus, Congenital Zika syndrome, Pediatrics, Hospitalization

## Abstract

**Objectives:**

Congenital Zika Syndrome (CZS) has been linked to a wide spectrum of abnormalities. However, differences in hospitalization patterns between children with and without CZS have not yet been investigated.

**Methods:**

We compared rates of hospital admissions for all and specific diseases, proportions of admission causes, and total length of hospital stay (LOS) between children with CZS and those without the syndrome. Adjusted incidence rate ratios (aIRR) and aLOS were estimated using negative binomial regression.

**Results:**

Compared to those without CZS, the aIRR for all-cause hospitalizations in the CZS group ranged from 3.77 (95%CI: 3.47-4.06) in the neonatal period to 7.76 (95%CI: 6.91-8.61) at ages 2-4 years. Similar trends were observed for specific causes of admissions. Most admissions in the CZS group related to congenital malformations, neurological, respiratory and infectious diseases. aLOS ranged from 16.0 days [95%CI: 13.2-19.5] to 19.9 days among CZS patients and 6.0 days [95%CI: 5.9-6.2] to 9.3 days [95%CI: 9.3-9.4] for patients without the syndrome.

**Conclusion:**

Children born with CZS face significantly higher rates of hospitalization and longer stays compared to those without the syndrome during early childhood.

## Highlights

• We evaluated almost 2000 children with confirmed or probable CZS and 2.6 million without CZS.
• We estimated hospitalization rates, causes, and length of stay for CZS and non-CZS
• Rates of hospitalization were 3 to 7 times higher among CZS vs non-CZS.
• Increased morbidity in CZS children persisted during the first 4 years of life.

## Introduction

Zika virus (ZIKV) is an arthropod-borne flavivirus that has had documented transmission in more than 80 countries and territories [1]. From 2015 to 2018, when the World Health Organization (WHO) declared Zika-related microcephaly a Public Health Emergency of International Concern [2], almost 600,000 suspected cases were reported and 223,477 confirmed only in the Americas [3]. The virus can cross the placental barrier, and it has a direct impact on the fetus neurogenesis, leading to adverse effects, such as the Congenital Zika syndrome (CZS) [[4], [5], [6]], which has been linked to a wide spectrum of structural anomalies (*e.g.*, microcephaly), functional impairments (*e.g.*, dysphagia, neurodevelopmental delays), neurological sequelae (*e.g.*, epilepsy), and increased mortality [4,[7], [8], [9], [10], [11]].

However, data on hospitalizations among children with CZS remains limited [12,13], and no studies have yet explored its differences in relation to children not affected by the syndrome. The present study aims to compare admission rates, their underlying causes, and length of stay between children with CZS and without CZS during their first 4 years of life.

Accurate information on the most common causes of hospitalization among children with CZS is crucial for guiding clinical practices, enabling tailored management for these patients, and assisting public health authorities in better estimating the healthcare resources needed in areas where ZIKV is endemic.

## Methods

### Data source

Information on the maternal-child dyads and pregnancy characteristics was obtained from the CIDACS (*Centro de Integração de Dados e Conhecimento para a Saúde*) Birth Cohort [14], which links records from both the Unified Registry for Social Programs (*Cadastro Único para Programas Sociais,* CADU), representing families who have applied for social assistance [15], and the Live Birth Information System (*Sistema de Informaçoes Sobre Nascidos Vivos,* SINASC), covering nearly all births in Brazil [16,17].

From the CIDACS Birth Cohort, data were retained about the mother (age, educational attainment, marital status, and race/ethnicity), the pregnancy (number of prenatal appointments, length of gestation, and status of having multiple fetuses), and the newborn (birth weight and sex).

Information about CZS was obtained from the Public Health Events Registry (*Registro de Eventos em Saúde Pública,* RESP) [18]. After notification, all suspected CZS cases were investigated by the epidemiologic surveillance teams and classified as confirmed, probable, inconclusive, or excluded. From this record, data on final CZS classification, microcephaly at birth, and presence of syphilis or toxoplasmosis infections were retained. See Additional Methods (Supplementary material) for further details.

Death-related information was obtained from the Mortality Information System (*Sistema de Informação de Mortalidade,* SIM) [19], which records data from death certificates (*i.e*., legal documents that must be completed by the physician responsible for clinical care, an assistant, or other practitioner from the institution who attests to the cause of death) for nearly 97% of deaths in Brazil.

Finally, information on hospital admissions were obtained from the hospital information system (*Sistema de Informações Hospitalares*, SIH) of the Brazilian Unified Health System (*Sistema Único de Saúde,* SUS) [20], which records data on hospital admissions, procedures performed, length of stay, International Classification of Diseases (ICD)-coded diagnoses, and other relevant information. It is estimated that this system covers 70% of all hospital admissions in Brazil, with potentially higher coverage among lower socioeconomic populations [21].

### Linkage process

The CIDACS Birth Cohort was linked with SIH, RESP, and SIM records using the variables of mother's name, date of birth or age, and residence applying the CIDACS-RL algorithm, a record linkage tool that was developed to link large scale administrative data sets from Brazil by a combination of indexing and searching-algorithms approaches [22]. Linkage procedures were conducted at CIDACS (Salvador, Bahia, Brazil) in a strict data-protection environment and according to ethical and legal rules.

### Study population

We included all singleton live births from the final linked dataset, born from January 1, 2015, to December 31, 2018.

**Figure 1:**
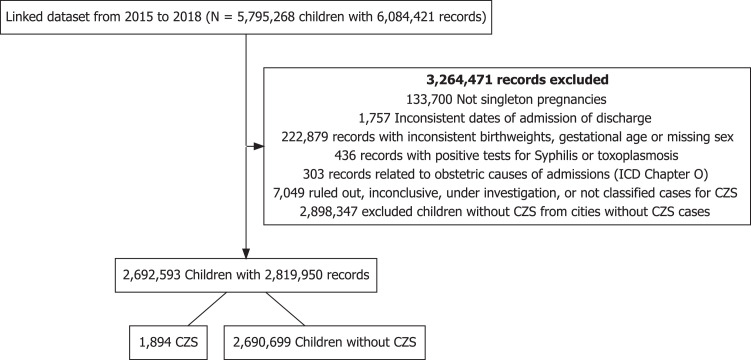
Flowchart of study population.

We excluded children with: (1) inconsistent hospital admission or discharge dates (*e.g.*, discharge occurring prior to admission or admission before date of birth); (2) birth weights of <500 g or >5000 g; (3) gestational ages of <22 weeks or >42 weeks; (4) with missing data for child sex; (5) hospital admissions related to obstetric causes; (6) a final CZS classification of inconclusive or excluded in RESP records; and, for children with CZS only, a positive test for either syphilis or toxoplasmosis, as recorded in RESP (Figure 1). Finally, we restricted the analysis to cities with at least one confirmed case of CZS to prevent sparse data bias.

### Exposure and outcome definitions

We considered cases of CZS only live births with a final classification on RESP as confirmed or probable CZS. Children without CZS were defined as all live births included in our cohort without a RESP registry.

Our primary outcomes were: (1) the number of SUS hospital admissions, stratified by age at admission, categorized as 0 to 27 days, 28 to 365 days, 1-2 years (from 366 days to 730 days), and 2-4 years (from 731 days to 1460 days); and (2) Primary admission causes, as recorded in the Authorization for Hospital Admission Request Form (AIH) according to the International Classification of Diseases, 10th Revision (ICD-10). Secondary causes were not included as they might reflect prevalent rather than incident conditions, therefore not directly responsible for the current admission. See Additional Methods (Supplementary material) for further details on AIH.

All hospital entries from birth to the age of 3 years, 11 months, and 29 days were included. To avoid counting transfers or admissions to a new hospital for the same disease as multiple hospitalizations, we collapsed new hospitalizations for the same ICD-10 code within 6 days of the previous hospitalization's discharge date.

Secondary outcomes included: (1) the number of hospital admissions due to four specific ICD-10 chapters, considered as important causes of hospitalization during early life - Certain infectious and parasitic diseases (A00-B99); Diseases of the nervous system (G00-G99); Diseases of the respiratory system – (J00-J99) and Diseases of the digestive system (K00-K93); (2) The total length of stay (LOS) at hospital, within each age range (0-365 days,1-2 years, and 2-4 years); (3) The number of hospital admissions and (4) LOS at hospital for CZS children with and without microcephaly.

### Statistical analysis

Descriptive statistics were used for exploring general characteristics of individuals with and without CZS. Crude hospital admission rates per 100 person years were calculated for both CZS and non-CZS groups, and stratified by age at admission (0-27 days, 28-365 days, 1-2 years, 2-4 years).

This procedure was performed first for all causes of hospital admissions, then for the specific causes. Cumulative LOS was calculated as the sum of the durations, in days, of all admissions occurring within each age range.

We then fitted a generalized linear model with a negative binomial distribution with person-years as an offset and a log link to estimate adjusted incidence rate ratios and 95% confidence intervals, first for all-cause hospital admissions and then for the specific ICD-10 chapters, comparing the CZS group to the non-CZS group. The same model was used to estimate adjusted LOS during each age range, in days, for both groups.

We adjusted for the following variables, considered as possible confounders of the association between CZS and hospitalization: maternal race/ethnicity (white or black/mixed/other), education (>7 or ≤ 7 years of study) and age (<20, 20-34, or ≥35 years old). We also adjusted for the state of residence (27 Brazilian states), except in the analysis of specific causes of hospitalization and for admission rate analysis among microcephalic children, where, in the latter case, region of residence was used, due to the small number of cases. Furthermore, we repeated the primary analysis (1) without any collapsed hospitalizations within 6 days and (2) including only confirmed cases of CZS (excluding probable cases) to test the consistency of our results.

The children were followed from date of birth until date of death, December 31, 2018 (final date of data collection) or completed 4 years of age, whichever occurred first. Missing data were excluded from analysis. Findings were considered statistically significant if the 95% CI did not cross the reference value of 1. All analyses were performed in R statistical software (version 3.6.0) [23].

## Results

The final dataset comprised 2,692,593 children, including 1894 (0.07%) individuals with suspected or confirmed CZS (*i.e*., of whom n = 796, 42.0% had microcephaly) and 2,690,699 individuals without the syndrome (Figure 1 and Table 1).

**Table 1:** Descriptive table—characteristics of study population.

Variables	Non-CZS (N = 2,690,699)	CZS cases (N = 1894)
**Sex**		
Male	1,377,988 (51.2%)	891 (47.0%)
Female	1,312,711 (48.8%)	1003 (53.0%)
**Birth weight (g)**		
<1500	29,374 (1.1%)	82 (4.3%)
1500-2499	174,460 (6.5%)	572 (30.2%)
≥2500	2,486,865 (92.4%)	1240 (65.5%)
**Gestational age at birth (weeks)**		
<32	37,086 (1.4%)	52 (2.7%)
32-36	239,175 (8.9%)	318 (16.8%)
≥37	2,414,438 (89.7%)	1524 (80.5%)
**Size for gestational age**^a^		
Adequate for gestational age	2,056,379 (76.4%)	1083 (57.2%)
Small for gestational age	204,946 (7.6%)	687 (36.3%)
Large for gestational age	429,374 (16.0%)	124 (6.5%)
**Birth year**		
2015	857,605 (31.9%)	834 (44.0%)
2016	618,679 (23.0%)	816 (43.1%)
2017	612,675 (22.8%)	164 (8.7%)
2018	601,740 (22.4%)	80 (4.2%)
**Mother age (years)**		
<20	554,529 (20.6%)	475 (25.1%)
20-34	1,852,346 (68.8%)	1238 (65.4%)
≥35	283,823 (10.5%)	181 (9.6%)
Missing	1 (0.0%)	0 (0.0%)
**Maternal race/ethnicity**		
White	507,738 (19%)	287 (15%)
Black	193,936 (7.2%)	116 (6.1%)
Yellow	8306 (0.3%)	4 (0.2%)
Brown	1,834,558 (68%)	1369 (72%)
Indigenous	14,304 (0.5%)	10 (0.5%)
Missing	131,857 (4.9%)	108 (5.7%)
**Marital status**		
Married or in civil union	1,157,084 (43.0%)	869 (45.9%)
Single, widowed, or divorced	1,509,681 (56.1%)	1002 (52.9%)
Missing	23,934 (0.9%)	23 (1.2%)
**Educational attainment**		
0-3 yr	78,441 (2.9%)	71 (3.7%)
4-7 yr	552,207 (20.5%)	457 (24.1%)
8-11 yr	1,803,750 (67.0%)	1199 (63.3%)
≥ 12yr	224,759 (8.4%)	141 (7.4%)
Missing	31,542 (1.2%)	26 (1.4%)
**Mode of delivery**		
Vaginal	1,440,459 (53.5%)	985 (52.0%)
Cesarean	1,248,949 (46.4%)	908 (47.9%)
Missing	1291 (0.0%)	1 (0.1%)
**No. of prenatal appointments**		
0	52,386 (1.9%)	56 (3.0%)
1-3	210,828 (7.8%)	162 (8.6%)
4-6	749,378 (27.9%)	564 (29.8%)
≥7	1,662,461 (61.8%)	1,097 (57.9%)
Missing	15,646 (0.6%)	15 (0.8%)
**Geographic region of Brazil**		
Southeast	991,380 (36.8%)	406 (21.4%)
South	77,724 (2.9%)	25 (1.3%)
Central west	243,893 (9.1%)	129 (6.8%)
Northeast	1,116,939 (41.5%)	1238 (65.4%)
North	260,763 (9.7%)	96 (5.1%)
**Age at first hospital admission (months)**	0 (0-7)	0 (0-8)
**Hospitalization**	560,983 (20.8%)	1259 (66.5%)
**Number of hospital admissions**		
0	2,129,716 (79.2%)	635 (33.5%)
1	487,742 (18.1%)	841 (44.4%)
2	48,229 (1.8%)	165 (8.7%)
3-4	19,099 (0.7%)	140 (7.4%)
>4	5913 (0.2%)	113 (6.0%)
**Total days in-hospital during study period (among hospitalized children)**	5 (3-10)	10 (5-23)
**Time of RESP notification (CZS)**		
Pregnancy/Fetal period	NA	47 (2.5%)
Neonatal period (< 28 days)	NA	1531 (80.8%)
Post-neonatal period (≥ 28 days)	NA	316 (16.7%)
**Microcephaly**	NA	796 (42.0%)
**Anomaly detection (including microcephaly)**		
Intrauterine	NA	466 (24.6%)
Post-partum	NA	988 (52.2%)
Not detected	NA	11 (0.6%)
Missing	NA	429 (22.6%)
**Death**	29,197 (1.1%)	188 (9.9%)
**Age at death (among children who died)**		
< 28 days	17,763 (60.8%)	72 (38.3%)
28-89 days	4387 (15.0%)	24 (12.8%)
90-364 days	4311 (14.8%)	58 (30.8%)
1-2 years	1718 (5.9%)	19 (10.1%)
2-4 years	1018 (3.5%)	15 (8.0%)

Data is represented as n (%) or median (p25-p75).

a Size for gestational age was defined based on intergrowth charts and comprised: (1) small for gestational age (SGA) – *i.e*. birth weight <10th percentile for sex and gestational age; (2) Appropriate for gestational age (AGA) – *i.e*. birthweight between 10th and 90th percentiles for sex and gestational age; (3) Large for gestational age (LGA) – *i.e*. birthweight > 90th percentile for sex and gestational age.

Compared to the group without the syndrome, the children with CZS had a higher incidence of preterm birth (19.5% vs 10.3%) and of being small for gestational age (36.3% vs 7.6%). Additionally, most children with CZS were born in the Northeast region (65.4%), in contrast to 41.5% among children without CZS.

During the follow-up period, 1259 (66.5%) children with CZS had at least one hospital admission compared to 560,983 (20.8%) without CZS. They also experienced longer hospital stays, with a median duration of 10 days (IQR: 5-23 days) during all the study period, compared to 5 days (IQR: 3-10 days) among children without the syndrome - Table 1 and Supplementary Figure 1. Mortality rates were also higher among CZS patients (188 - 9.9% vs 29,197 - 1.1%), with most deaths in both groups occurring within the first year of life (82% vs 91%).

### Primary outcomes

Table 2 presents crude hospital admission rates per 100 person-years, categorized by the age of children at admission. Across all age groups, the incidence rate was higher for children with CZS compared to those without CZS, ranging from 28.5 (2-4 years) to 576.4 (0-27 days) hospital admissions per 100 person years for CZS patients, compared to 3.5 (2-4 years) to 163.2 (0-27 days) admissions per 100 person years for non-CZS.

**Table 2:** Descriptive characteristics of hospital admissions (for all causes) for CZS and non-CZS children, by age at admission.

	Non-CZS				CZS cases			
Age categories	N deaths	N admissions	Person-years	Rate^a^	N deaths	N admissions	Person-years	Rate^a^
0-27 days	17,763	320,618	196,474.4	163.2	72	781	135.5	576.4
28-365 days	8703	209,465	2,173,327.2	9.6	82	682	1605.2	42.5
1-2 years	1719	100,837	1,767,315.0	5.7	19	638	1600.1	39.9
2-4 years	1012	56,196	1,601,996.5	3.5	15	384	1347.4	28.5

a Rate represents the number of hospital admissions per 100 person-years. Data included 1894 CZS and 2,690,699 controls.

**Figure 2:**
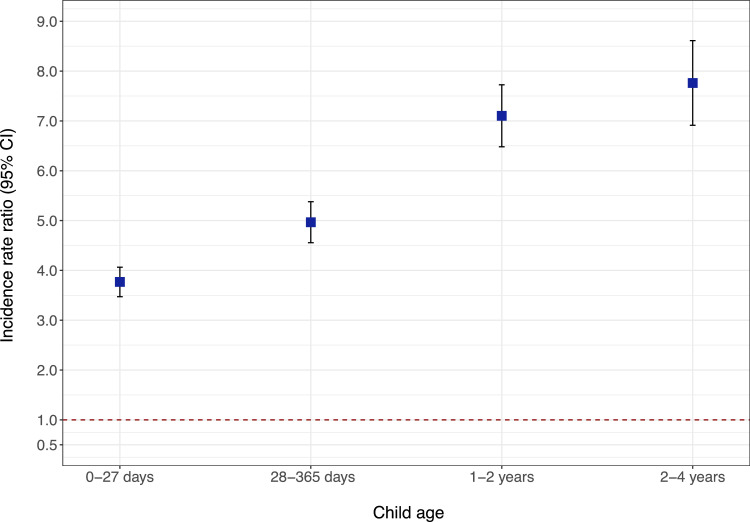
Adjusted incidence rate ratios and 95% CI for all-cause hospital admissions for CZS compared to children without CZS by age at admission. Point estimates and error bars represent the incidence rate ratios (IRRs) and their 95% confidence intervals for hospital admissions due to any cause, comparing children with CZS to children without CZS (reference group).

As age increases, we observed a greater decrease in hospital admission rates for those without CZS than for CZS patients. Figure 2 and Supplementary Table 1 display the adjusted incidence rate ratios (IRR) for all-cause hospitalizations by age at admission. The IRR for the entire study period was 4.77 (95% CI, 4.53-5.02), ranging from 3.77 (95% CI, 3.47-4.06) during the neonatal period (0-27 days) to a peak at 7.76 (95% CI, 6.91-8.61) at ages 2-4 years.

**Figure 3:**
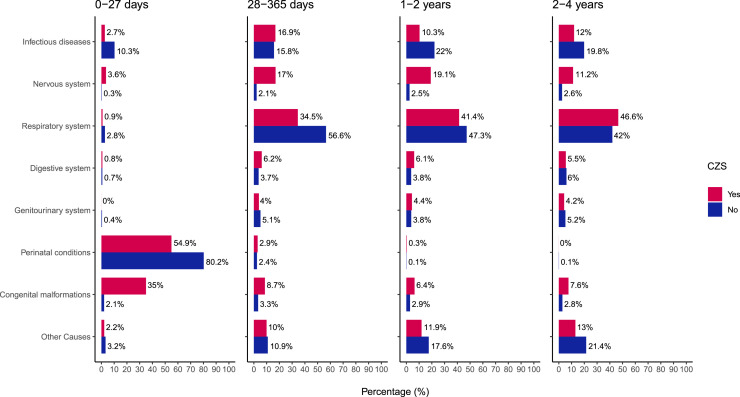
Specific causes of admissions by ICD-10 Chapters among children with and without CZS stratified by age at admission. Data and percentages refer to 2485 admissions among the CZS group and 687,116 admissions among the non-CZS group during all the follow up period (January 1, 2015 to December 31, 2018); ICD-10 Codes - Infectious diseases (A00-B99); Nervous system (G00-G99); Respiratory system (J00-J99); Digestive system (K00-K93); Genitourinary system (N00-N99); Perinatal conditions (P00-P96); Congenital malformations (Q00-Q99).

During all age periods, the CZS group had more admissions related to diseases of the nervous system, and congenital malformations compared to children without the syndrome. In contrast, the reference group was more often admitted due to conditions originating in the perinatal period, as well as to infectious, parasitic, and respiratory system diseases (Figure 3). As children with CZS aged, neurological diseases remained significant reasons for hospitalizations, and the prevalence of respiratory and infectious causes progressively increased (Supplementary Figure 2 and Supplementary Table 2).

### Secondary outcomes

After restricting the analysis to the four specific ICD-10 chapter codes, hospital admission rates tended to decrease with increased ages (from 0-1 years to 2-4 years), for both groups, but the decreases were more pronounced for non-CZS children (Certain infectious and parasitic diseases [CZS: 7.79-3.41 vs non-CZS: 2.78-0.70]; Diseases of the nervous system [8.25-3.19 vs 0.22-0.09]; Diseases of the digestive system [2.75-1.56 vs 0.42-0.21]) - Supplementary Table 3.

For diseases of the respiratory system, rates among CZS children remained high during all age periods, reaching approximately 17 admissions per 100 person-years at 1-2 years old, almost 8-times higher than rate for children without CZS of the same age. Supplementary Figure 3 displays the corresponding adjusted IRRs with 95% CIs, by age at admission.

Across all ages, children with CZS tended to be hospitalized for more days compared to non-CZS patients. During the first year of life, they had a cumulative LOS of 19.9 days (95% CI, 17.9-22.1) compared to 9.4 days (95% CI, 9.3-9.4) for children without CZS.

Although both groups experienced a reduction in LOS at older ages, the decrease was less pronounced for CZS patients. At 2-4 years old, their LOS remained almost three times longer than that of the comparison group (16.6 days [95% CI, 11.9 - 23.2] vs 6.0 days [95% CI, 5.9 - 6.2]) - Supplementary Table 4.

Supplementary Table 5 displays general characteristics of CZS children by the presence of microcephaly. Compared to normocephalic children with CZS, microcephalic patients were more frequently small-for gestational age (38.3% vs 34.8%), had mothers of white race (18.8% vs 12.5%), were born by vaginal delivery (55.7% vs 49.4%) and more frequently from north region (6.8% vs 3.8%). Also, they had a lower mortality rate (7.2% vs 11.9%), with 75% of them dying before the first year of life, compared to 85% of children without microcephaly.

Concerning hospital admissions, CZS patients with microcephaly displayed a lower rate of hospital admission within the first year but similar rates for 1-2 and 2-4 years compared to children without microcephaly. After adjustment for region of residence, IRR were significantly lower among microcephalic children only at 0-365 days (0.71 [95% CI, 0.57-0.85]) - Supplementary Tables 6 and 7. Regarding admission causes and total LOS at hospital, no significant differences were observed between both groups (Supplementary Figure 4 and Supplementary Table 8).

After repeating primary analysis without collapsing hospitalizations within 6 days, we observed no changes in the general trends of our findings (Supplementary Table 9). Finally, general characteristics of CZS children based on final classification criteria (Confirmed vs Probable) are presented in Supplementary Table 10. After restricting the analysis to only confirmed CZS cases, our main findings were reinforced (Supplementary Table 11 and Supplementary Table 12).

## Discussion

In this population-based cohort study, which included over 2.6 million children from Brazil, CZS was associated with 3 to 7-fold increases in hospital admission rates and persistently longer length of hospital stays during the first 4 years of life.

Unlike the comparison group, the decrease in hospitalization rates with aging in children with CZS was much smaller, exhibiting persistently high incidence rate ratios (IRRs). Additionally, causes of hospital admissions differed between groups and varied with increasing ages, with most admissions in the CZS group related to congenital malformations, neurological diseases alongside respiratory and infectious causes. Finally, our results suggested the increased rates were independent of the presence of microcephaly.

Some studies have demonstrated increased frequencies of medical visits among CZS patients. A retrospective case series conducted in two Brazilian states with follow-up of 24 months, observed that approximately 63% of patients consulted in the emergency room during the study period [12]. Another retrospective study conducted in the state of Salvador (Brazil) has shown that near 49% of CZS children were admitted at least once during the first 2 years of life, with 15% of admissions being related to invasive procedures, like orthopedic surgery, gastrostomy, tracheostomy, neurosurgical procedures, and gastroesophageal reflux surgery [13]. In both studies, respiratory infections and diseases of the nervous system were the leading causes of hospitalization.

Supporting these findings, we observed consistently elevated IRRs for hospital admissions due to neurologic disorders among children under 4 years with CZS, which is likely explained by congenital alterations of the central nervous system, predisposing children to seizures, motor function impairments, and delayed neurodevelopment [[24], [25], [26], [27], [28], [29]]. Additionally, respiratory, digestive, and infectious causes were also significantly higher among CZS children during all age intervals. This underscores the double burden of disease they face, as their baseline neurological impairments and congenital malformations coexist with common childhood illnesses, such as respiratory and infectious diseases.

In this scenario, each condition can act as a risk factor for the other (*e.g.* aspiration pneumonia from dysphagia, infections from invasive procedures like tracheostomy or gastrostomy tube), increasing the overall morbidity of affected children.

Some studies have found that the increased risks of adverse outcomes among CZS children are not fully explained by the presence of microcephaly, as normocephalic patients are also at increased risks of delayed neurodevelopment [30,31]. This was reinforced by our findings, as we observed similar hospitalization rates for both groups. However, the unexpectedly lower admission rate during the first year for children with microcephaly as well as the increased mortality rate among normocephalic CZS patients may reflect a selection bias.

Microcephaly is often associated with miscarriages, so restricting the analysis to live-born microcephalic children may have led to the inclusion of less severe cases. Moreover, the broader case definitions at the beginning of the pandemic could also have included children with microcephaly but no other severe abnormalities. Additionally, mild normocephalic CZS cases may be underdiagnosed compared to patients with abnormal head circumferences, and at the time of clinical suspicion, they may present with other congenital malformations that might be as severe as those affecting microcephalic children. This is supported by similar proportions of small-for-gestational-age and preterm births in both groups.

Finally, an aspect worth noting refers to the increased costs reported for CZS care, as our findings suggested this group also stays hospitalized for longer periods. Two studies conducted in Brazil estimated costs incurred by the healthcare system and patients’ families due to CZS [32,33]. The cost per child over 10 years for health providers ranged from US$ 5000 to US$ 16,000 for CZS children depending on the severity of disease, whereas for controls, it was estimated a cost of US$2,400 for the same period. Additionally, nearly 30% of families of children with CZS reported caring costs that exceed 40% of their yearly household income. These findings reinforce the importance of incorporating hospitalization data into healthcare planning for children with CZS, ensuring that resources are appropriately allocated to address their unique health needs.

Our study has several strengths. We used a nationwide linked cohort, with high sample size, including more than 1800 confirmed and probable CZS cases that were notified in the country and had the possibility of being admitted into a public hospital.

However, our results must be interpreted with caution due to some limitations. First, we only included children that met CZS specific case definition from RESP. This might have excluded children that meet more recent case definitions, retaining the most severe cases of the disease, and possibly overestimating their hospitalization rates. Additionally, although the SIH database has high coverage, it does not capture private hospital entries, which could result in missing some admissions. However, our study population consists of only children registered on the CIDACS Birth Cohort, representing a socio-economically vulnerable population unlikely to have private health insurance or afford direct hospital costs.

We also lacked information on other infectious diseases known to cause congenital neurological symptoms, such as cytomegalovirus, herpes, and rubella. However, congenital herpes is known to be rare, most congenital cytomegalovirus infections are asymptomatic and not routinely screened during antenatal care, and no cases of congenital rubella syndrome have been confirmed in Brazil since 2010 [34]. Together, these factors, along with the sensitivity analysis comparing confirmed and probable cases, reduce the potential impact of these conditions on our findings.

Finally, as an administrative data, SIH might record multiple ICD-10 codes for the same admission (*e.g.*, new diagnoses made during admission), and restricting the analysis to only primary causes of hospitalization might impose further limitations to define the exact number of hospital entries a child had or its underlying cause. Despite that, our sensitivity analysis changing the threshold of days for defining two consecutive entries as different hospitalizations did not change the trend of our results.

## Conclusions

The present study found that live-born children with Congenital Zika syndrome have persistently higher rates of hospitalization compared to those without the syndrome during their first 4 years of life. These findings highlight the substantial disease burden and associated healthcare costs for affected children, carrying important implications for families, healthcare providers, and policymakers, particularly in regions where Zika virus is endemic.

## Declaration of competing interest

The authors have no competing interests to declare.

## Appendix Supplementary materials

Image, application 1
