# Supplementary material for: Association between congenital Zika syndrome and hospitalizations during early childhood: a nationwide cohort study
Source: Int J Infect Dis. 2025 Apr;153:None. doi: 10.1016/j.ijid.2025.107780 (PMC11910342; doi:10.1016/j.ijid.2025.107780)
Supplement: Supplementary file 1 [file mmc1.docx]

**SUPPLEMENTARY MATERIAL**

**Title:** Association between Congenital Zika Syndrome and hospitalizations during early childhood: a nationwide cohort study

**Authors:** João Guilherme G. Tedde, MD, Thiago Cerqueira Silva, MD/Ph.D, Laura Rodrigues, MD/Ph.D, Maria da Conceição Costa, Ph.D, Luciana Cardim, Ph.D, Elizabeth B. Brickley, Ph.D, Maria Gloria Teixeira, Ph.D, Mauricio L. Barreto, MD/Ph.D, Enny S. Paixão, Ph.D

**Table of Contents**

[**Additional Methods** 1](#_Toc185500961)

[**Supplementary Figure 1 - Distribution of total days in hospital during all the follow-up period for children with and without CZS** 2](#_Toc185500962)

[**Supplementary Table 1 - Adjusted incidence rate ratios (IRR) and 95% CI for all-cause hospital admissions for CZS patients compared to non-CZS children (reference) by age at admission.** 3](#_Toc185500963)

[**Supplementary Figure 2 - Specific causes of admissions by ICD-10 Chapters among CZS and non-CZS children stratified by age at admission.** 4](#_Toc185500964)

[**Supplementary Table 2 - Proportions of ICD-10 Chapters among CZS and Non-CZS children stratified by age at admission.** 5](#_Toc185500965)

[**Supplementary Table 3 - Comparative characteristics of hospital admissions between CZS and non-CZS children, stratified by ICD-10 chapter and age at admission.** 8](#_Toc185500966)

[**Supplementary Figure 3 - Adjusted incidence rate ratios and 95% CI for specific causes of hospital admissions for CZS compared to non-CZS children by age at admission.** 9](#_Toc185500967)

[**Supplementary Table 4 - Adjusted length of stay (LOS) in days for CZS and non-CZS children, stratified by age at admission** 9](#_Toc185500968)

[**Supplementary Table 5 – Descriptive table of CZS children, according to the presence of microcephaly** 10](#_Toc185500969)

[**Supplementary Table 6 – Descriptive characteristics of all-cause hospital admissions among CZS group, by age at admission and presence of microcephaly.** 13](#_Toc185500970)

[**Supplementary Table 7 - Adjusted incidence rate ratios of all-cause hospital admissions among CZS group, by age at admission and presence of microcephaly.** 13](#_Toc185500971)

[**Supplementary Figure 4 - Specific causes of admissions by ICD-10 Chapters among CZS according to the presence of microcephaly.** 14](#_Toc185500972)

[**Supplementary Table 8 – Adjusted length of stay (LOS) in days for CZS children, stratified by age at admission and presence of microcephaly.** 14](#_Toc185500973)

[**Supplementary Table 9 - Adjusted incidence rate ratios (IRR) and 95% CI for all-cause hospital admissions for CZS patients compared to children without CZS (reference) by age at admission. Sensitivity analysis without any collapsed hospitalizations within 6 days.** 15](#_Toc185500974)

[**Supplementary Table 10 - Descriptive table of CZS children, according to the final classification.** 15](#_Toc185500975)

[**Supplementary Table 11 - Adjusted incidence rate ratios (IRR) and 95% CI for all-cause hospital admissions for CZS patients compared to children without CZS (reference) by age at admission. Analysis restricted to confirmed CZS.** 19](#_Toc185500976)

[**Supplementary Table 12 - Adjusted length of stay (LOS) in days for CZS and non-CZS children, stratified by age at admission. Analysis restricted to confirmed CZS.** 19](#_Toc185500977)

[**References** 19](#_Toc185500978)

**Additional Methods**

**Additional information on RESP system**

Since 2015, the *Registro de Eventos em Saúde Pública* (RESP) system has recorded information about all suspected cases of CZS in Brazil. Suspected cases of CZS include live-born children who present with one or more of the following criteria: (1) Microcephaly detected during pregnancy or at birth (i.e., defined as a head circumference of more than 2 SD below the mean for age and sex); (2) Craniofacial disproportion; (3) Central nervous system changes suggestive of congenital infection as detected on neuroimaging tests; (4) Two or more neurologic, visual, or auditory manifestations suggestive of congenital infection; (5) birth from a mother who reported prenatal fever and/or rash, which were suspected or confirmed to be related to a ZIKV infection during pregnancy. Image findings suggestive of CZS can be found in the Brazilian Ministry of Health Guideline.[1] Suspected cases were subjected to complete investigation and classified as follows:


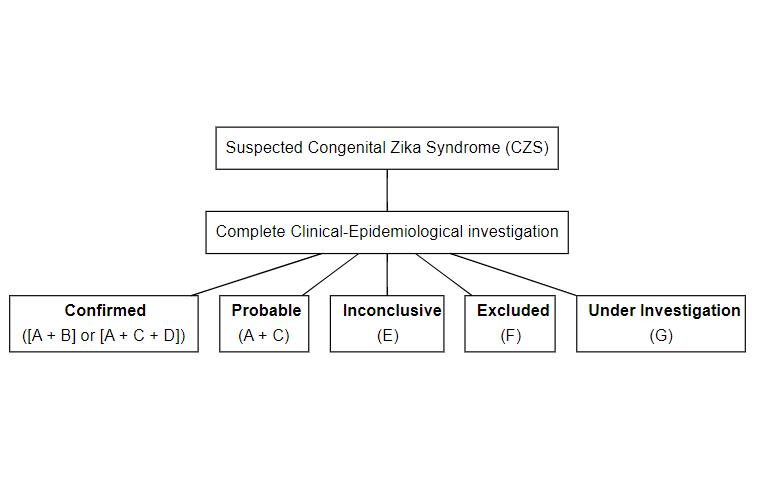


**Legend:** **A** – Two or more signs and symptoms suggestive of congenital zika syndrome by clinical examination or image; **B** – Positive RT-PCR result for ZIKV infection or serological test of a sample of the mother, fetus, or newborn (in the first 48 hours of life); **C** – Negative/Inconclusive/Unavailable results for RT-PCR or serological tests for ZIKV infection from samples of the mother, fetus, or newborn (in the first 48 hours of life); **D** – Maternal report of rash or fever during pregnancy; **E** – Clinical-Epidemiological investigation not completed due to refusal or to impossibility to contact after three or more attempts by surveillance teams; **F** – Diagnosis of another etiology or final classification not compatible with definitions of confirmed, probable or inconclusive cases after clinical-epidemiological investigation; **G** – Clinical-Epidemiological investigation so far had not yet reached enough information to enable diagnostic classification.

**Additional information on AIH**

The *Sistema de Informações Hospitalares* (SIH) records both primary and secondary causes of hospitalization. The primary cause would be the one considered, through clinical judgment, to be the primary responsible for each admission and the other contributing causes would be recorded as secondary causes. However, if a new diagnosis is detected during the same hospitalization and it requires additional care or procedures, a new AIH (Authorization for Hospital Admission—the legal document required for all public hospitalizations) will be issued with the new diagnosis as the primary cause. Additional information can be found in the SIH guideline [2]. It is important to note that before October 2018, a specific ICD-10 code for CZS (P35.4) did not exist. Consequently, we might expect that a proportion of hospitalizations during the neonatal period to have been classified under categories other than "Certain conditions originating in the perinatal period - (P00-P96)” among children with CZS.

**Supplementary Figure 1 - Distribution of total days in hospital during all the follow-up period for children with and without CZS**


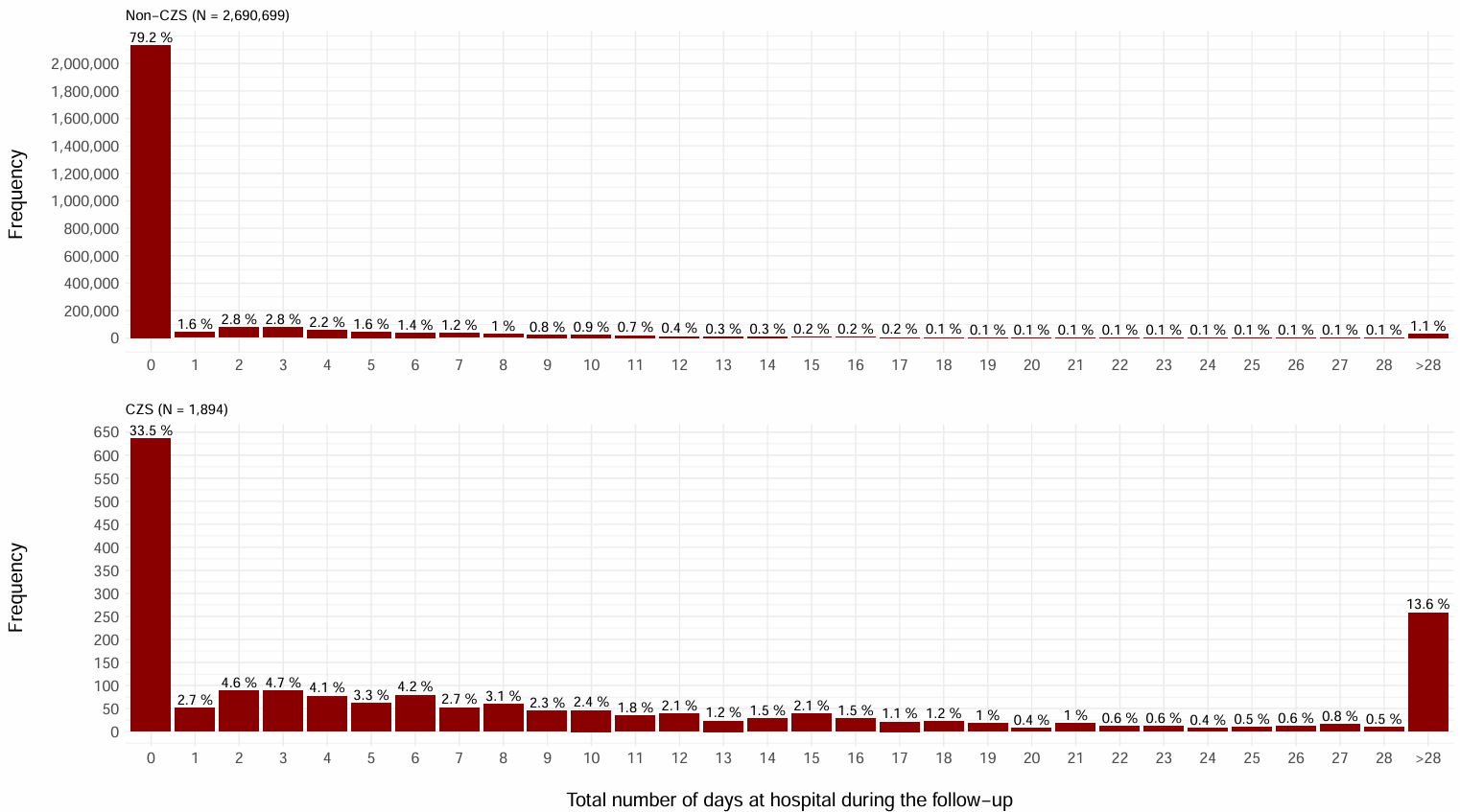


**Supplementary Table 1 - Adjusted incidence rate ratios (IRR) and 95% CI for all-cause hospital admissions for CZS patients compared to non-CZS children (reference) by age at admission.**

|  | **CZS** | | |
| --- | --- | --- | --- |
| **Age categories** | **IRR** | **95% CI** | |
| Overall (0-4 years) | 4.77 | 4.53 | 5.02 |
| 0-27 days | 3.77 | 3.47 | 4.06 |
| 28 – 365 days | 4.97 | 4.55 | 5.38 |
| 1-2 years | 7.10 | 6.48 | 7.72 |
| 2-4 years | 7.76 | 6.91 | 8.61 |
| Adjusted for maternal race/ethnicity, maternal education, maternal age, and state of residence. | | | |

**Supplementary Figure 2 - Specific causes of admissions by ICD-10 Chapters among CZS and non-CZS children stratified by age at admission.**


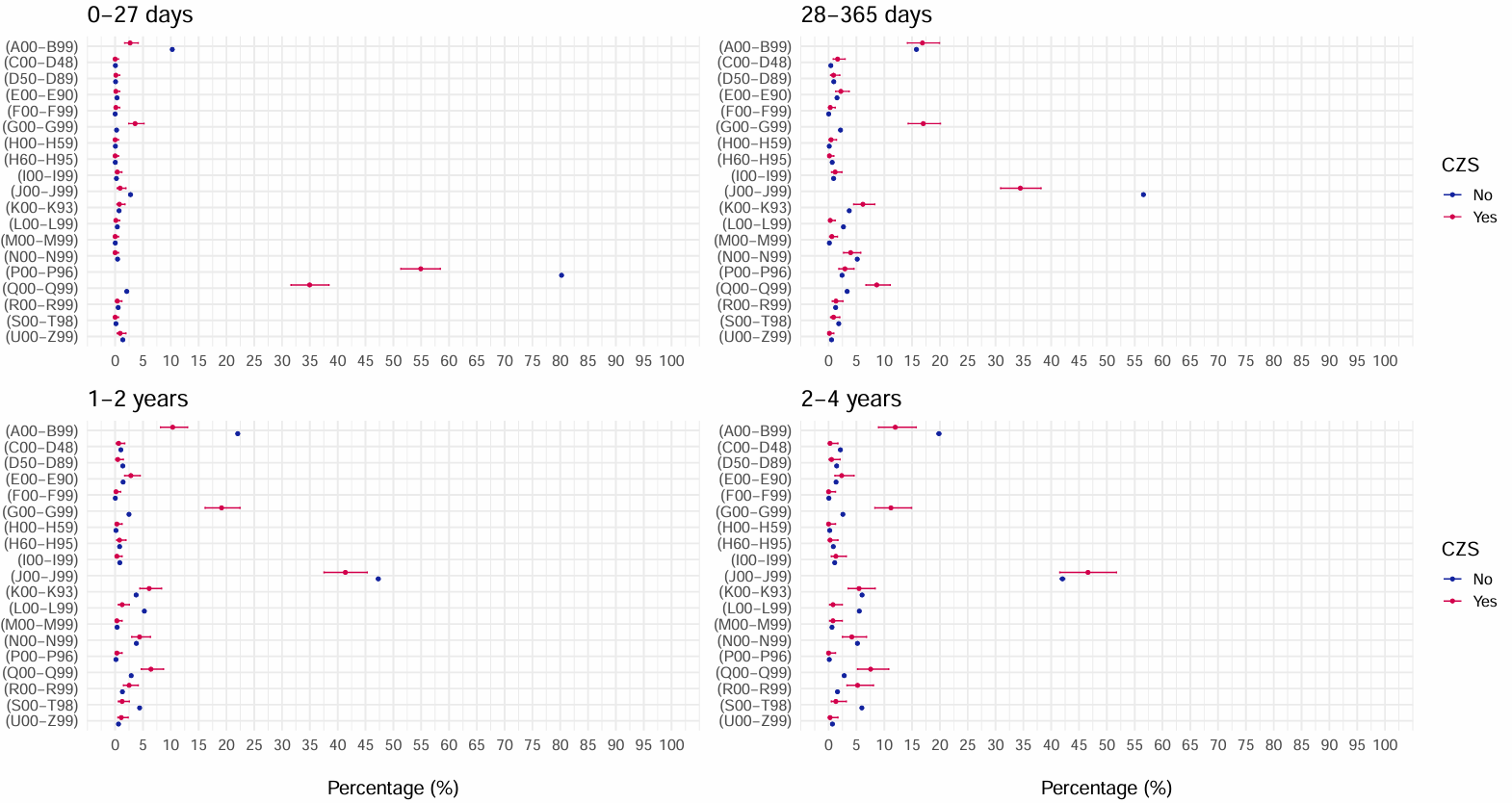


Supplementary Figure 2 legend: Data shows proportions and their 95% CI for ICD-10 codes related to admissions.

**Supplementary Table 2 - Proportions of ICD-10 Chapters among CZS and Non-CZS children stratified by age at admission.**

|  | |  | **Non-CZS** | | | | **CZS** | | | | |
| --- | --- | --- | --- | --- | --- | --- | --- | --- | --- | --- | --- |
| **ICD Chapter** | **0-27 days** | | | **28-365 days** | **1-2 years** | **2-4 years** | | **0-27 days** | **28-365 days** | **1-2 years** | **2-4 years** |
| Certain infectious and parasitic diseases (A00-B99) | 32,930 (10.27% [10.17-10.38]) | | | 33,051 (15.78% [15.62-15.94]) | 22,219 (22.03% [21.78-22.29]) | 11,145 (19.83% [19.5-20.16]) | | 21 (2.69% [1.71-4.15]) | 115 (16.86% [14.17-19.93]) | 66 (10.34% [8.14-13.04]) | 46 (11.98% [8.99-15.75]) |
| Neoplasms (C00-D48) | 150 (0.05% [0.04-0.06]) | | | 829 (0.4% [0.37-0.42]) | 1,035 (1.03% [0.97-1.09]) | 1,189 (2.12% [2-2.24]) | | 0 (0% [0-0.61]) | 11 (1.61% [0.85-2.96]) | 4 (0.63% [0.2-1.71]) | 1 (0.26% [0.01-1.67]) |
| Diseases of the blood and blood-forming organs and certain disorders involving the immune mechanism (D50-D89) | 245 (0.08% [0.07-0.09]) | | | 1,936 (0.92% [0.88-0.97]) | 1,388 (1.38% [1.31-1.45]) | 811 (1.44% [1.35-1.55]) | | 1 (0.13% [0.01-0.83]) | 6 (0.88% [0.36-2.01]) | 3 (0.47% [0.12-1.49]) | 2 (0.52% [0.09-2.08]) |
| Endocrine, nutritional and metabolic diseases (E00-E90) | 1,064 (0.33% [0.31-0.35]) | | | 3,161 (1.51% [1.46-1.56]) | 1,439 (1.43% [1.36-1.5]) | 752 (1.34% [1.25-1.44]) | | 1 (0.13% [0.01-0.83]) | 15 (2.2% [1.28-3.69]) | 18 (2.82% [1.73-4.51]) | 9 (2.34% [1.15-4.56]) |
| Mental and behavioral disorders (F00-F99) | 9 (0% [0-0.01]) | | | 34 (0.02% [0.01-0.02]) | 32 (0.03% [0.02-0.05]) | 24 (0.04% [0.03-0.06]) | | 1 (0.13% [0.01-0.83]) | 2 (0.29% [0.05-1.18]) | 1 (0.16% [0.01-1.01]) | 0 (0% [0-1.23]) |
| Diseases of the nervous system (G00-G99) | 837 (0.26% [0.24-0.28]) | | | 4,470 (2.13% [2.07-2.2]) | 2,507 (2.49% [2.39-2.58]) | 1,443 (2.57% [2.44-2.7]) | | 28 (3.59% [2.44-5.21]) | 116 (17.01% [14.31-20.09]) | 122 (19.12% [16.19-22.44]) | 43 (11.2% [8.31-14.89]) |
| Diseases of the eye and adnexa (H00-H59) | 144 (0.04% [0.04-0.05]) | | | 244 (0.12% [0.1-0.13]) | 150 (0.15% [0.13-0.18]) | 110 (0.2% [0.16-0.24]) | | 0 (0% [0-0.61]) | 3 (0.44% [0.11-1.39]) | 2 (0.31% [0.05-1.26]) | 0 (0% [0-1.23]) |
| Diseases of the ear and mastoid process (H60-H95) | 88 (0.03% [0.02-0.03]) | | | 1,353 (0.65% [0.61-0.68]) | 811 (0.8% [0.75-0.86]) | 474 (0.84% [0.77-0.92]) | | 0 (0% [0-0.61]) | 1 (0.15% [0.01-0.95]) | 5 (0.78% [0.29-1.93]) | 1 (0.26% [0.01-1.67]) |
| Diseases of the circulatory system (I00-I99) | 737 (0.23% [0.21-0.25]) | | | 1,879 (0.9% [0.86-0.94]) | 846 (0.84% [0.78-0.9]) | 616 (1.1% [1.01-1.19]) | | 3 (0.38% [0.1-1.22]) | 8 (1.17% [0.55-2.39]) | 2 (0.31% [0.05-1.26]) | 5 (1.3% [0.48-3.19]) |
| Diseases of the respiratory system (J00-J99) | 8,861 (2.76% [2.71-2.82]) | | | 118,512 (56.58% [56.37-56.79]) | 47,687 (47.29% [46.98-47.6]) | 23,618 (42.03% [41.62-42.44]) | | 7 (0.9% [0.39-1.92]) | 235 (34.46% [30.92-38.18]) | 264 (41.38% [37.54-45.32]) | 179 (46.61% [41.56-51.74]) |
| Diseases of the digestive system (K00-K93) | 2,256 (0.7% [0.68-0.73]) | | | 7,765 (3.71% [3.63-3.79]) | 3,808 (3.78% [3.66-3.9]) | 3,380 (6.01% [5.82-6.22]) | | 6 (0.77% [0.31-1.75]) | 42 (6.16% [4.53-8.3]) | 39 (6.11% [4.44-8.34]) | 21 (5.47% [3.5-8.37]) |
| Diseases of the skin and subcutaneous tissue (L00-L99) | 1,255 (0.39% [0.37-0.41]) | | | 5,576 (2.66% [2.59-2.73]) | 5,297 (5.25% [5.12-5.39]) | 3,099 (5.51% [5.33-5.71]) | | 1 (0.13% [0.01-0.83]) | 2 (0.29% [0.05-1.18]) | 8 (1.25% [0.58-2.56]) | 3 (0.78% [0.2-2.46]) |
| Diseases of the musculoskeletal system and connective tissue (M00-M99) | 78 (0.02% [0.02-0.03]) | | | 291 (0.14% [0.12-0.16]) | 354 (0.35% [0.32-0.39]) | 341 (0.61% [0.55-0.68]) | | 0 (0% [0-0.61]) | 4 (0.59% [0.19-1.6]) | 2 (0.31% [0.05-1.26]) | 3 (0.78% [0.2-2.46]) |
| Diseases of the genitourinary system (N00-N99) | 1,402 (0.44% [0.41-0.46]) | | | 10,781 (5.15% [5.05-5.24]) | 3,845 (3.81% [3.7-3.93]) | 2,912 (5.18% [5-5.37]) | | 0 (0% [0-0.61]) | 27 (3.96% [2.67-5.78]) | 28 (4.39% [2.99-6.36]) | 16 (4.17% [2.48-6.82]) |
| Certain conditions originating in the perinatal period (P00-P96) | 257,244 (80.23% [80.1-80.37]) | | | 5,064 (2.42% [2.35-2.48]) | 149 (0.15% [0.13-0.17]) | 69 (0.12% [0.1-0.16]) | | 429 (54.93% [51.36-58.45]) | 20 (2.93% [1.85-4.57]) | 2 (0.31% [0.05-1.26]) | 0 (0% [0-1.23]) |
| Congenital malformations, deformations and chromosomal abnormalities (Q00-Q99) | 6,683 (2.08% [2.04-2.13]) | | | 6,969 (3.33% [3.25-3.4]) | 2,913 (2.89% [2.79-2.99]) | 1,576 (2.8% [2.67-2.95]) | | 273 (34.96% [31.63-38.43]) | 59 (8.65% [6.7-11.08]) | 41 (6.43% [4.7-8.69]) | 29 (7.55% [5.2-10.78]) |
| Symptoms, signs and abnormal clinical and laboratory findings, not elsewhere classified (R00-R99) | 1,753 (0.55% [0.52-0.57]) | | | 2,639 (1.26% [1.21-1.31]) | 1,313 (1.3% [1.23-1.37]) | 893 (1.59% [1.49-1.7]) | | 3 (0.38% [0.1-1.22]) | 9 (1.32% [0.65-2.58]) | 16 (2.51% [1.49-4.13]) | 20 (5.21% [3.29-8.06]) |
| Injury, poisoning and certain other consequences of external causes (S00-T98) | 467 (0.15% [0.13-0.16]) | | | 3,810 (1.82% [1.76-1.88]) | 4,435 (4.4% [4.27-4.53]) | 3,358 (5.98% [5.78-6.18]) | | 0 (0% [0-0.61]) | 6 (0.88% [0.36-2.01]) | 8 (1.25% [0.58-2.56]) | 5 (1.3% [0.48-3.19]) |
| Other Causes (U00-Z99) | 4,413 (1.38% [1.34-1.42]) | | | 1,101 (0.53% [0.5-0.56]) | 609 (0.6% [0.56-0.65]) | 386 (0.69% [0.62-0.76]) | | 7 (0.9% [0.39-1.92]) | 1 (0.15% [0.01-0.95]) | 7 (1.1% [0.48-2.35]) | 1 (0.26% [0.01-1.67]) |
| Data is presented as N (% [95% CI]) | | | | | | | | | | | |

**Supplementary Table 3 - Comparative characteristics of hospital admissions between CZS and non-CZS children, stratified by ICD-10 chapter and age at admission.**

| **Certain infectious and parasitic diseases (A00-B99)** | | | | | | |
| --- | --- | --- | --- | --- | --- | --- |
|  | **Non-CZS** | | | **CZS** | | |
| **Age categories** | **N admissions** | **Person-years** | **Rate*** | **N admissions** | **Person-years** | **Rate*** |
| 0-1 year | 65,981 | 2,377,011.1 | 2.78 | 136 | 1,745.7 | 7.79 |
| 1-2 years | 22,219 | 1,767,314.9 | 1.26 | 66 | 1,600.1 | 4.12 |
| 2-4 years | 11,145 | 1,601,996.5 | 0.70 | 46 | 1,347.4 | 3.41 |
| **Diseases of the nervous system (G00-G99)** | | | | | | |
|  | **Non-CZS** | | | **CZS** | | |
| **Age categories** | **N admissions** | **Person-years** | **Rate*** | **N admissions** | **Person-years** | **Rate*** |
| 0-1 year | 5,307 | 2,377,011.1 | 0.22 | 144 | 1,745.7 | 8.25 |
| 1-2 years | 2,507 | 1,767,314.9 | 0.14 | 122 | 1,600.1 | 7.62 |
| 2-4 years | 1,443 | 1,601,996.5 | 0.09 | 43 | 1,347.4 | 3.19 |
| **Diseases of the digestive system (K00-K93)** | | | | | | |
|  | **Non-CZS** | | | **CZS** | | |
| **Age categories** | **N admissions** | **Person-years** | **Rate*** | **N admissions** | **Person-years** | **Rate*** |
| 0-1 year | 10,021 | 2,377,011.1 | 0.42 | 48 | 1,745.7 | 2.75 |
| 1-2 years | 3,808 | 1,767,314.9 | 0.21 | 39 | 1,600.1 | 2.44 |
| 2-4 years | 3,380 | 1,601,996.5 | 0.21 | 21 | 1,347.4 | 1.56 |
| **Diseases of the respiratory system – ICD 10 J00-J99** | | | | | | |
|  | **Non-CZS** | | | **CZS** | | |
| **Age categories** | **N admissions** | **Person-years** | **Rate*** | **N admissions** | **Person-years** | **Rate*** |
| 0-1 year | 127,373 | 2,377,011.1 | 5.36 | 242 | 1,745.7 | 13.86 |
| 1-2 years | 47,687 | 1,767,314.9 | 2.70 | 264 | 1,600.1 | 16.50 |
| 2-4 years | 23,618 | 1,601,996.5 | 1.47 | 179 | 1,347.4 | 13.28 |
| *Rate represents the number of hospital admissions per 100 person-years. | | | | | | |

**Supplementary Figure 3 - Adjusted incidence rate ratios and 95% CI for specific causes of hospital admissions for CZS compared to non-CZS children by age at admission.**


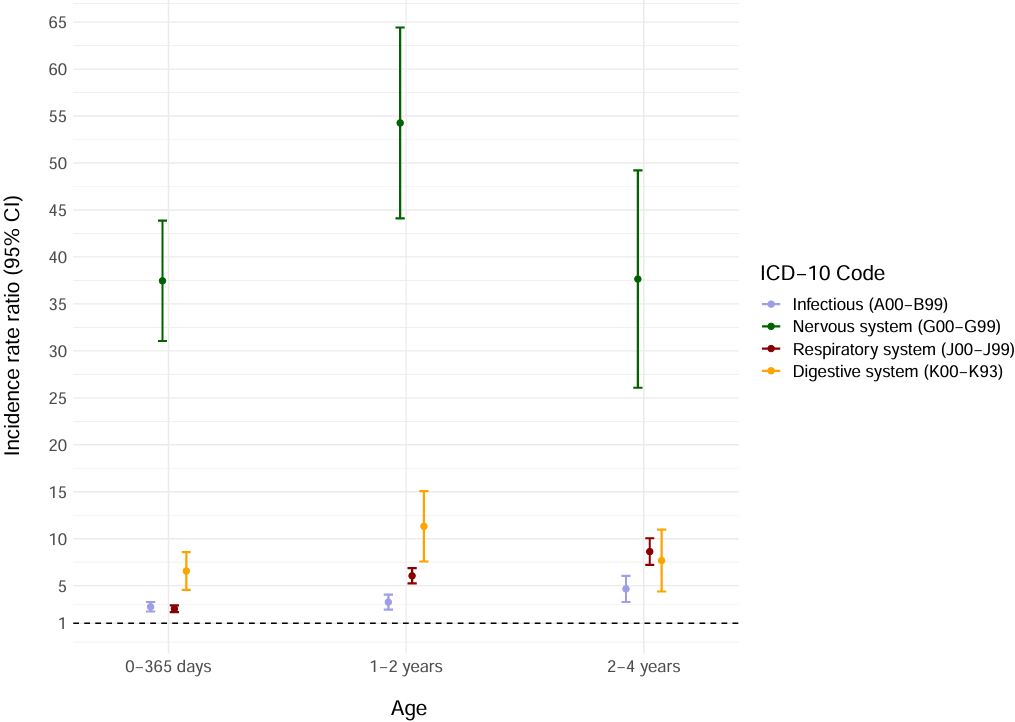


Supplementary Figure 3 legend: Adjusted for maternal race/ethnicity, maternal education, maternal age, and region of residence.

**Supplementary Table 4 - Adjusted length of stay (LOS) in days for CZS and non-CZS children, stratified by age at admission**

|  | **Non-CZS** | | **CZS** | |
| --- | --- | --- | --- | --- |
| **Age** | **LOS** | **95% CI** | **LOS** | **95% CI** |
| 0-365 days | 9.38 | 9.34 – 9.43 | 19.92 | 17.91 – 22.15 |
| 1-2 years | 6.46 | 6.36 – 6.57 | 16.05 | 13.20 – 19.51 |
| 2-4 years | 6.05 | 5.90 – 6.20 | 16.63 | 11.92 – 23.20 |

Adjusted for maternal race/ethnicity, maternal education, maternal age, and state of residence.

**Supplementary Table 5 – Descriptive table of CZS children, according to the presence of microcephaly**

| **Variable** | **Normocephaly,**  **N = 1,098** | **Microcephaly,**  **N = 796** |
| --- | --- | --- |
| **Sex** |  |  |
| Male | 518 (47.2%) | 373 (46.9%) |
| Female | 580 (52.8%) | 423 (53.1%) |
| **Birth weight (g)** |  |  |
| <1500 | 47 (4.3%) | 35 (4.4%) |
| 1500-2499 | 328 (29.9%) | 244 (30.7%) |
| ≥2500 | 723 (65.8%) | 517 (64.9%) |
| **Gestational age at birth (weeks)** |  |  |
| <32 | 34 (3.1%) | 18 (2.3%) |
| 32-36 | 192 (17.5%) | 126 (15.8%) |
| ≥37 | 872 (79.4%) | 652 (81.9%) |
| **Size for Gestational Age ^a^** |  |  |
| Adequate for gestational age | 628 (57.2%) | 455 (57.2%) |
| Small for gestational age | 382 (34.8%) | 305 (38.3%) |
| Large for gestational age | 88 (8.0%) | 36 (4.5%) |
| **Birth Year** |  |  |
| 2015 | 466 (42.4%) | 368 (46.2%) |
| 2016 | 477 (43.4%) | 339 (42.6%) |
| 2017 | 102 (9.3%) | 62 (7.8%) |
| 2018 | 53 (4.8%) | 27 (3.4%) |
| **Mother age (years)** |  |  |
| <20 | 266 (24.2%) | 209 (26.3%) |
| 20-34 | 727 (66.2%) | 511 (64.2%) |
| ≥35 | 105 (9.6%) | 76 (9.5%) |
| **Maternal race/ethnicity** |  |  |
| black/mixed/other | 892 (81.2%) | 607 (76.3%) |
| white | 137 (12.5%) | 150 (18.8%) |
| Missing | 69 (6.3%) | 39 (4.9%) |
| **Marital status** |  |  |
| Married or in civil union | 497 (45.3%) | 372 (46.7%) |
| Single, widowed, or divorced | 587 (53.5%) | 415 (52.1%) |
| Missing | 14 (1.3%) | 9 (1.1%) |
| **Educational attainment** |  |  |
| 0-3 yr | 47 (4.3%) | 24 (3.0%) |
| 4-7 yr | 262 (23.9%) | 195 (24.5%) |
| 8-11 yr | 688 (62.7%) | 511 (64.2%) |
| ≥ 12yr | 82 (7.5%) | 59 (7.4%) |
| Missing | 19 (1.7%) | 7 (0.9%) |
| **Mode of delivery** |  |  |
| Vaginal | 542 (49.4%) | 443 (55.7%) |
| Cesarean | 555 (50.5%) | 353 (44.3%) |
| Missing | 1 (0.1%) | 0 (0.0%) |
| **No. of prenatal appointments** |  |  |
| 0 | 37 (3.4%) | 19 (2.4%) |
| 1-3 | 89 (8.1%) | 73 (9.2%) |
| 4-6 | 329 (30.0%) | 235 (29.5%) |
| ≥7 | 631 (57.5%) | 466 (58.5%) |
| Missing | 12 (1.1%) | 3 (0.4%) |
| **Geographic region of Brazil** |  |  |
| Southeast | 233 (21.2%) | 173 (21.7%) |
| South | 10 (0.9%) | 15 (1.9%) |
| Central west | 73 (6.6%) | 56 (7.0%) |
| Northeast | 740 (67.4%) | 498 (62.6%) |
| North | 42 (3.8%) | 54 (6.8%) |
| **Hospitalization** | 742 (67.6%) | 517 (64.9%) |
| **Age at first hospital admission (months)** | 0.1 (0.0 – 6.5) | 0.1 (0.0 – 8.9) |
| **Number of hospital admissions** |  |  |
| 0 | 356 (32.4%) | 279 (35.1%) |
| 1 | 493 (44.9%) | 348 (43.7%) |
| 2 | 97 (8.8%) | 68 (8.5%) |
| 3-4 | 90 (8.2%) | 50 (6.3%) |
| >4 | 62 (5.6%) | 51 (6.4%) |
| **Total days in-hospital during study period (among hospitalized children)** | 10.5 (4.0 – 26.0) | 10.0 (5.0 – 21.0) |
| **Death** | 131 (11.9%) | 57 (7.2%) |
| **Age at death (among children who died)** |  |  |
| <1 mo | 53 (40.4%) | 20 (35.1%) |
| [1-3[ mo | 16 (12.2%) | 7 (12.3%) |
| [3-6[ mo | 18 (13.7%) | 7 (12.3%) |
| [6-9[ mo | 15 (11.4%) | 3 (5.3%) |
| [9-12[ mo | 9 (6.9%) | 6 (10.5%) |
| [12-24[ mo | 10 (7.6%) | 9 (15.8%) |
| [24-36[ mo | 9 (6.9%) | 5 (8.8%) |
| [36-48[ mo | 1 (0.8%) | 0 (0.0%) |

Data is represented as n (%) or median (p25 – p75);

^a^ Size for gestational age was defined based on intergrowth charts and comprised: (1) small for gestational age (SGA) – i.e. birth weight <10th percentile for sex and gestational age; (2) Appropriate for gestational age (AGA) – i.e. birthweight between 10th and 90th percentiles for sex and gestational age; (3) Large for gestational age (LGA) – i.e. birthweight > 90th percentile for sex and gestational age.

**Supplementary Table 6 – Descriptive characteristics of all-cause hospital admissions among CZS group, by age at admission and presence of microcephaly.**

|  | | | **Normocephaly,**  **N = 1098** | | | **Microcephaly,**  **N = 796** | | | |
| --- | --- | --- | --- | --- | --- | --- | --- | --- | --- |
| **Age categories** | **N**  **Admissions** | **Person-years** | **Rate*** | **N Deaths** | **N**  **Admissions** | **Person-years** | **Rate*** | **N Deaths** |  |
| 0-365 days | 899 | 991.8 | 90.6 | 111 | 564 | 749.0 | 75.3 | 43 |  |
| 1-2 years | 348 | 903.1 | 38.5 | 10 | 290 | 697.0 | 41.6 | 9 |  |
| 2-4 years | 220 | 772.9 | 28.5 | 10 | 164 | 574.4 | 28.6 | 5 |  |
| *Rate represents the number of hospital admissions per 100 person-years. | | | | | | | | | |

**Supplementary Table 7 - Adjusted incidence rate ratios of all-cause hospital admissions among CZS group, by age at admission and presence of microcephaly.**

|  | **Microcephaly** | | |
| --- | --- | --- | --- |
| **Age categories** | **IRR** | **95% CI** | |
| 0-365 days | 0.71 | 0.57 | 0.85 |
| 1-2 years | 0.99 | 0.60 | 1.37 |
| 2-4 years | 1.01 | 0.80 | 1.21 |
| Adjusted rate ratios refer to microcephalic CZS children compared to normocephalic (reference group); the model was adjusted for region of residence. | | | |

**Supplementary Figure 4 - Specific causes of admissions by ICD-10 Chapters among CZS according to the presence of microcephaly.**

**
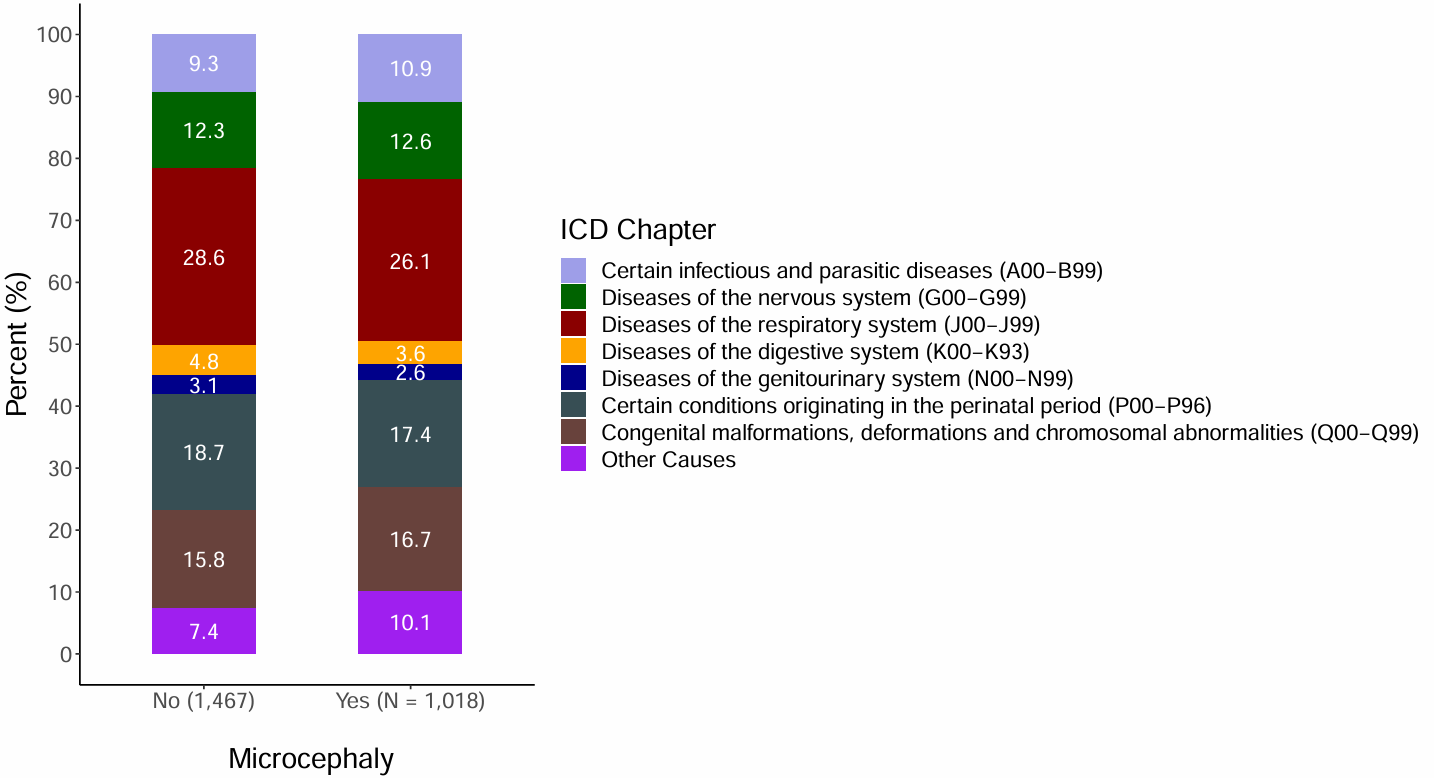
**

Supplementary Figure 4 legend: Data refers to the number of admissions during all the study period.

**Supplementary Table 8 – Adjusted length of stay (LOS) in days for CZS children, stratified by age at admission and presence of microcephaly.**

|  | **Normocephaly** | | **Microcephaly** | |
| --- | --- | --- | --- | --- |
| **Age** | **LOS** | **95% CI** | **LOS** | **95% CI** |
| 0-365 days | 18.9 | 16.6 – 21.4 | 16.7 | 13.8 – 20.3 |
| 1-2 years | 16.5 | 12.6 – 21.5 | 11.9 | 9.3 – 15.3 |
| 2-4 years | 15.7 | 11.5 – 21.4 | 11.6 | 8.3 – 16.2 |
| Adjusted for maternal race/ethnicity, maternal education, maternal age, and region of residence. | | | | |

**Supplementary Table 9 - Adjusted incidence rate ratios (IRR) and 95% CI for all-cause hospital admissions for CZS patients compared to children without CZS (reference) by age at admission. Sensitivity analysis without any collapsed hospitalizations within 6 days.**

|  | **CZS** | | |
| --- | --- | --- | --- |
| **Age categories** | **IRR** | **95% CI** | |
| 0-28 days | 4.31 | 3.93 | 4.69 |
| 29 – 365 days | 5.07 | 4.60 | 5.54 |
| 1-2 years | 6.61 | 5.98 | 7.24 |
| 2-4 years | 7.06 | 6.24 | 7.88 |
| Adjusted for maternal ethnicity, maternal education, maternal age, and state of residence. | | | |

**Supplementary Table 10 - Descriptive table of CZS children, according to the final classification.**

|  | **CZS** | |
| --- | --- | --- |
| **Variable** | **Confirmed,**  **N = 1,565** | **Probable,**  **N = 329** |
| **Sex** |  |  |
| Male | 737.0 (47.1%) | 154.0 (46.8%) |
| Female | 828.0 (52.9%) | 175.0 (53.2%) |
| **Birth weight (g)** |  |  |
| <1500 | 62.0 (4.0%) | 20.0 (6.1%) |
| 1500-2499 | 472.0 (30.2%) | 100.0 (30.4%) |
| ≥2500 | 1,031.0 (65.9%) | 209.0 (63.5%) |
| **Gestational age at birth (weeks)** |  |  |
| <32 | 38.0 (2.4%) | 14.0 (4.3%) |
| 32-36 | 264.0 (16.9%) | 54.0 (16.4%) |
| ≥37 | 1,263.0 (80.7%) | 261.0 (79.3%) |
| **Size for Gestational Age ^a^** |  |  |
| Adequate for gestational age | 905.0 (57.8%) | 178.0 (54.1%) |
| Small for gestational age | 567.0 (36.2%) | 120.0 (36.5%) |
| Large for gestational age | 93.0 (5.9%) | 31.0 (9.4%) |
| **Birth Year** |  |  |
| 2015 | 765.0 (48.9%) | 69.0 (21.0%) |
| 2016 | 681.0 (43.5%) | 135.0 (41.0%) |
| 2017 | 78.0 (5.0%) | 86.0 (26.1%) |
| 2018 | 41.0 (2.6%) | 39.0 (11.9%) |
| **Mother age (years)** |  |  |
| <20 | 401.0 (25.6%) | 74.0 (22.5%) |
| 20-34 | 1,028.0 (65.7%) | 210.0 (63.8%) |
| ≥35 | 136.0 (8.7%) | 45.0 (13.7%) |
| **Maternal race/ethnicity** |  |  |
| black/mixed/other | 1,253.0 (80.1%) | 246.0 (74.8%) |
| white | 225.0 (14.4%) | 62.0 (18.8%) |
| Missing | 87.0 (5.6%) | 21.0 (6.4%) |
| **Marital status** |  |  |
| Married or in civil union | 723.0 (46.2%) | 146.0 (44.4%) |
| Single, widowed, or divorced | 823.0 (52.6%) | 179.0 (54.4%) |
| Missing | 19.0 (1.2%) | 4.0 (1.2%) |
| **Educational attainment** |  |  |
| 0-3 yr | 61.0 (3.9%) | 10.0 (3.0%) |
| 4-7 yr | 385.0 (24.6%) | 72.0 (21.9%) |
| 8-11 yr | 983.0 (62.8%) | 216.0 (65.7%) |
| ≥ 12yr | 117.0 (7.5%) | 24.0 (7.3%) |
| Missing | 19.0 (1.2%) | 7.0 (2.1%) |
| **Mode of delivery** |  |  |
| Vaginal | 842.0 (53.8%) | 143.0 (43.5%) |
| Cesarean | 722.0 (46.1%) | 186.0 (56.5%) |
| Missing | 1.0 (0.1%) | 0.0 (0.0%) |
| **No. of prenatal appointments** |  |  |
| 0 | 39.0 (2.5%) | 17.0 (5.2%) |
| 1-3 | 129.0 (8.2%) | 33.0 (10.0%) |
| 4-6 | 468.0 (29.9%) | 96.0 (29.2%) |
| ≥7 | 920.0 (58.8%) | 177.0 (53.8%) |
| Missing | 9.0 (0.6%) | 6.0 (1.8%) |
| **Geographic region of Brazil** |  |  |
| Southeast | 265.0 (16.9%) | 141.0 (42.9%) |
| South | 20.0 (1.3%) | 5.0 (1.5%) |
| Central west | 109.0 (7.0%) | 20.0 (6.1%) |
| Northeast | 1,079.0 (68.9%) | 159.0 (48.3%) |
| North | 92.0 (5.9%) | 4.0 (1.2%) |
| **Hospitalization** | 1,042.0 (66.6%) | 217.0 (66.0%) |
| **Age at first hospital admission (months)** | 0.1 (0.0, 8.9) | 0.0 (0.0, 2.2) |
| **Number of hospital admissions** |  |  |
| 0 | 523.0 (33.4%) | 112.0 (34.0%) |
| 1 | 698.0 (44.6%) | 143.0 (43.5%) |
| 2 | 137.0 (8.8%) | 28.0 (8.5%) |
| 3-4 | 116.0 (7.4%) | 24.0 (7.3%) |
| >4 | 91.0 (5.8%) | 22.0 (6.7%) |
| **Total days in-hospital during study period (among hospitalized children)** | 9.0 (4.0 - 22.0) | 14.0 (6.0 - 27.0) |
| **Death** | 139.0 (8.9%) | 49.0 (14.9%) |
| **Age at death (among children who died)** |  |  |
| < 28 days | 52.0 (37.4%) | 20.0 (40.8%) |
| 28-89 days | 19.0 (13.7%) | 5.0 (10.2%) |
| 90-364 days | 39.0 (28.1%) | 19.0 (38.8%) |
| 1-2 years | 16.0 (11.5%) | 3.0 (6.1%) |
| 2-4 years | 13.0 (9.4%) | 2.0 (4.1%) |
| Data is represented as n (%) or median (p25 – p75);  ^a^ Size for gestational age was defined based on intergrowth charts and comprised: (1) small for gestational age (SGA) – i.e. birth weight <10th percentile for sex and gestational age; (2) Appropriate for gestational age (AGA) – i.e. birthweight between 10th and 90th percentiles for sex and gestational age; (3) Large for gestational age (LGA) – i.e. birthweight > 90th percentile for sex and gestational age. | | |

**Supplementary Table 11 - Adjusted incidence rate ratios (IRR) and 95% CI for all-cause hospital admissions for CZS patients compared to children without CZS (reference) by age at admission. Analysis restricted to confirmed CZS.**

|  | **CZS** | | |
| --- | --- | --- | --- |
| **Age categories** | **IRR** | **95% CI** | |
| 0-28 days | 3.67 | 3.36 | 4.00 |
| 29 – 365 days | 4.58 | 4.15 | 5.01 |
| 1-2 years | 7.32 | 6.64 | 8.00 |
| 2-4 years | 8.07 | 7.16 | 8.98 |
| Adjusted for maternal ethnicity, maternal education, maternal age, and state of residence. | | | |

**Supplementary Table 12 - Adjusted length of stay (LOS) in days for CZS and non-CZS children, stratified by age at admission. Analysis restricted to confirmed CZS.**

|  | **Non-CZS** | | **CZS** | |
| --- | --- | --- | --- | --- |
| **Age** | **LOS** | **95% CI** | **LOS** | **95% CI** |
| 0-365 days | 9.38 | 9.34 – 9.43 | 18.72 | 16.59 – 21.12 |
| 1-2 years | 6.46 | 6.36 – 6.57 | 16.14 | 13.01 – 20.01 |
| 2-4 years | 6.05 | 5.90 – 6.20 | 16.78 | 11.80 – 23.85 |

Adjusted for maternal race/ethnicity, maternal education, maternal age, and state of residence.

**References**

**[1]** Saúde M da. Orientações integradas de vigilância e atenção à saúde no âmbito da emergência de saúde pública de importância nacional. Ms; 2016.

**[2]** Departamento De Regulação AEC. Manual Técnico Do Sistema De Informação Hospitalar. Ministério da Saúde; 2007.
